# Intraoperative radiotherapy (IORT) of early breast cancer with low-energy x-rays in breast-conserving surgery: Prospective identification of pre- and intraoperative factors influencing the feasibility of IORT

**DOI:** 10.1007/s00066-023-02149-8

**Published:** 2023-10-04

**Authors:** Anja Grimm, Eva Wollmann, Elena Sperk, Christel Weiß, Marc Sütterlin, Sebastian Berlit, Benjamin Tuschy

**Affiliations:** 1 Department of Obstetrics and Gynecology, University Medical Center Mannheim, Medical Faculty Mannheim, Heidelberg University, Heidelberg, Germany; 2 Department of Radiation Oncology, University Medical Center Mannheim, Medical Faculty Mannheim, Heidelberg University, Heidelberg, Germany; 3 Mannheim Cancer Center, University Medical Center Mannheim, Medical Faculty Mannheim, Heidelberg University, Heidelberg, Germany; 4 Department of Medical Statistics and Biomathematics, Medical Faculty Mannheim, Heidelberg University, Heidelberg, Germany; 5 Department of Obstetrics and Gynecology, University Medical Center Mannheim, Theodor-Kutzer Ufer 1-3, Mannheim, Germany

**Keywords:** Breast neoplasm, Intrabeam, Boost, Accelerated partial breast irradiation (APBI), External beam radiotherapy (EBRT)

## Abstract

**Purpose:**

The aim of this study is to identify pre- and intraoperative factors indicating the feasibility of intraoperative radiotherapy (IORT) during breast-conserving surgery (BCS).

**Materials and methods:**

From January 2018 to December 2019, a total of 128 women undergoing BCS due to early breast cancer were included in this prospective observational study, independent of whether IORT was planned or not. Patient and tumor characteristics as well as surgical parameters that could potentially influence the feasibility of IORT were recorded for the entire collective. In addition, a preoperative senological assessment was performed and analyzed to assess the feasibility of IORT. Logistic regression was then used to identify relevant preoperative parameters and to generate a formula predicting the feasibility of IORT.

**Results:**

Of the 128 included women undergoing BCS, 46 were preoperatively rated to be feasible, 20 to be questionably feasible for IORT. Ultimately, IORT was realized in 30 patients. The most frequent reasons for omission of IORT were insufficient tumor-to-skin distance and/or an excessively large tumor cavity. Small clinical tumor size and large tumor-to-skin distance according to preoperative ultrasound were significantly related to accomplishment of IORT.

**Conclusion:**

We observed that preoperative ultrasound-based tumor–skin distance is a significant factor in addition to already known parameters to predict feasibility of IORT. Based on our findings we developed a formula to optimize IORT planning which might serve as an additional tool to improve patient selection for IORT in early breast cancer.

## Introduction

Breast cancer is the most common malignant tumor in women worldwide with a lifetime risk of approximately 12% in western countries [1, 2]. In early breast cancer, tumor excision during breast-conserving surgery (BCS) usually in combination with sentinel lymph-node biopsy is the standard treatment complemented with chemotherapy, targeted therapy, and/or endocrine therapy according to national guidelines [3]. After BCS whole-breast external beam radiotherapy (EBRT) represents the inevitable standard treatment for patients with early breast cancer in the context of adjuvant radiotherapy [3, 4].

Another therapeutic approach is accelerated partial breast irradiation (APBI), in which only a part of the breast is irradiated in a shorter timeframe compared to standard EBRT. Techniques used for APBI include balloon brachytherapy, multicatheter technique, external beam accelerated partial-breast irradiation and intraoperative radiotherapy with low energy x‑rays (IORT), providing comparable local control as well as an excellent cosmetic outcome compared to EBRT [5–11]. For electron intraoperative radiotherapy (ELIOT), long-term results of the ELIOT trial confirm higher rates of ipsilateral breast tumor recurrence (IBTR) in the ELIOT group than in the group receiving whole breast irradiation, without any differences in overall survival [12]. ELIOT should be offered to selected patients at low-risk of IBTR, which is reflected in the current guidelines for the use of APBI according to the American Society for Radiation Oncology (ASTRO) and the Groupe Européen de Curiétherapie-European Society for Therapeutic Radiology and Oncology (GEC-ESTRO) consensus statements [13, 14].

In terms of IORT with low energy x‑rays using the INTRABEAM® system (Carl Zeiss Meditec, Oberkochen, Germany), the results from the TARGIT‑A trial showed noninferiority of IORT in comparison to standard EBRT regarding regional recurrence, breast cancer mortality and overall survival [11, 15, 16]. IORT as a targeted therapy can also limit undesirable side effects, especially in terms of heart, lung, and skin toxicity [17]. Other benefits include less pain and better quality of life compared to EBRT [17]. According to the TARGIT‑A trial, no differences were shown in the two groups regarding the secondary endpoint wound infections, whereas grade 3 and 4 toxicities were significantly lower in the experimental arm. In a selected patient population with early breast cancer, IORT as the only radiotherapeutic procedure may shorten or completely replace the external irradiation that usually follows surgery and is therefore convenient for the patients [10]. This might also be helpful to shorten treatment time during a pandemic [18]. However, IORT as APBI to this point of time is not considered standard treatment [4].

Furthermore, IORT may also be used to apply an additional dose (boost) to the tumor bed in combination with EBRT. In addition to whole breast irradiation IORT with 50 kV x-rays as anticipated tumor bed boost with 12–20 Gy reduces the risk of local recurrence in the breast [19, 20] and is accepted as standard treatment in most countries, e.g., Germany [21].

Since IORT constitutes an interdisciplinary therapeutic approach generating additional temporal and logistic effort perioperatively, preoperative patient selection and accurate planning are essential. Due to this reason, suitability for and feasibility of IORT continue to be the focus of scientific interest and are matter of ongoing debate.

Based on existing literature, current guidelines dealing with IORT patient selection take predominantly demographic and histologic parameters (e.g., nodal status, multicentricity/multifocality, surgical margins) as well as tumor size into account: consensus statements for patient selection to provide IORT as APBI exist from the European Society for Radiotherapy and Oncology (ESTRO) [13], the American Society for Radiation Oncology (ASTRO) [14], the American Society of Breast Surgeons (ASBS) [22], and the German Society of Radiation Oncology (DEGRO) [23]. Sperk et al. could show that depending on which statement is chosen, these recommendations lead to a strongly varying number of patients classified as suitable for IORT: in a patient collective of 1108 women with early breast cancer, IORT was applicable in 34.2% as APBI according to the ESTRO and in 15.8% according to the ASTRO statement [24].

Beyond the existing guidelines, there is uncertainty in the selection of patients eligible for IORT. Preoperative diagnostic and surgical parameters and the general eligibility of women suffering from early breast cancer have been sparsely investigated, although IORT relies on adequate surgical management. In a retrospective study, 18.5% of planned IORT procedures were not performed due to various reasons that were only identified intraoperatively [25].

Due to this uncertainty about precise preoperative patient selection, the aim of our study was to prospectively investigate additional parameters targeting the actual feasibility of IORT under real-life conditions by means of preoperative diagnostic and intraoperative anatomical circumstances in order to potentially optimize clinical management for IORT in early breast cancer surgery.

## Materials and methods

Between January 2018 and December 2019, a total of 128 women undergoing BCS for early breast cancer were prospectively included in this observational study at the University Medical Center Mannheim, Heidelberg University, Germany. Participation in this investigation was voluntary; written informed consent was obtained from each woman upon recruitment. The study was conducted according to the guidelines of the Declaration of Helsinki and approved by the Ethics Committee II of the University of Heidelberg, Medical Faculty Mannheim (2018-501N-MA). According to the inclusion criteria, the study recruited women with early breast cancer aged 18–100 years, diagnosed by needle biopsy and suitable for wide local excision. The inclusion in this study was independent of whether an IORT was planned or not.

The main purpose was to collect a representative sample of clinical data from women with early breast cancer undergoing BCS to determine which patients (apart from existing selection criteria) were actually eligible for IORT as APBI or boost.

A clinical breast examination was carried out as a standardized procedure in every woman including inspection, palpation, and sonographic assessment of both breasts. The sonographic assessment included the size of the tumor in three dimensions, the tumor-to-skin distance, and the distance of the tumor to the nipple areola complex. In every woman an individual preoperative assessment regarding the feasibility for IORT (feasible/not feasible/questionable) was performed by the attending physician of the breast unit by using a standardized questionnaire (see Appendix, Table 6) regardless of whether the criteria for IORT were met according to current guidelines. For clinical evaluation for feasibility of IORT, there were no prespecified selection criteria in terms of tumor–skin distance or tumor size. For each patient, the surgical procedure was discussed individually in an interdisciplinary tumor board.

In addition, individual patient characteristics (e.g., age, menopause status, body mass index, bra size, breast density, pre-existing diseases, pre-surgery/-irradiation in the target area) and tumor characteristics (e.g., tumor biology, tumor size, nodal status, grading, hormone receptor status, uni-/multifocality) as well as surgical parameters (e.g., tumor-to-skin distance intraoperatively, size of the tumor cavity according to intraoperative estimation, surgical technique: segment resection, central segment resection, skin spindle removal) potentially influencing the accomplishment of IORT were assessed.

IORT was performed using the INTRABEAM® device (Carl Zeiss Surgical, Oberkochen, Germany). This system consists of a miniaturized linear accelerator emitting low-energy x‑ray photons (max. 50 kV), various spherical applicators, a floor stand, and a control unit. The surgical and radiotherapeutic procedures were performed in a standardized way in accordance with hospital and national protocols [23, 26–28]. The majority of our patients received IORT according to the TARGIT‑C trial with the corresponding inclusion criteria (≥ 50 years, cT1 or T2 ≤ 3.5 cm, cN0, cM0, invasive ductal carcinoma, positive hormone receptor status; in the absence of any risk factors: multifocality/multicentricity, extensive intraductal component [EIC, L1, N1] [29]), and some patients received IORT as a tumor bed boost prior to EBRT.

In case of an intraoperatively omitted IORT, the reasons for the omission of IORT were documented by the attending physician after surgery.

All statistical calculations were accomplished with SAS software, release 9.4 (SAS Institute Inc., Cary, NC, USA). For qualitative factors, absolute and relative frequencies are given. Quantitative variables are presented as mean ± standard deviation or as median together with the range.

Concerning statistical analyses, the following tests were used: 2 sample t‑test to compare two means when data were approximately normally distributed, Mann and Whitney’s U‑test for ordinally scaled or quantitative variables that were not normally distributed, Cochran–Armitage trend test for ordinally scaled characteristics with few categories, Chi^2^ (Χ^2^) test for qualitative characteristics and Fisher’s exact test, if the requirements of the Χ^2^ test were not met (for frequencies less than 5 to be expected under the null hypothesis).

For the outcome of the preoperative assessment IORT (feasible/not feasible/questionable), 2‑group comparisons were performed for each parameter possibly associated with the outcome (e.g., patient/tumor characteristics, perioperative factors). For this purpose, the “IORT unfeasible” and “questionably feasible” were combined into one subgroup. To analyze the outcome “IORT successfully accomplished”, again 2‑group comparisons were performed to find a statistically association between the characteristics (e.g., patient/tumor characteristics, perioperative factors) and the target variable.

Furthermore, for the outcome “IORT feasible” (yes/no or questionable), a multiple logistic regression analysis was performed to generate a statistical model in which multiple variables were analyzed simultaneously. AUC (area under the curve) has been calculated in order to assess the goodness of the model. In general, a test result has been regarded as statistically significant for *p* < 0.05.

## Results

A total of 128 women who were treated for early breast cancer with BCS from January 2018 to December 2019 at University Medical Center Mannheim met the inclusion criteria. Women in our study population were on average 62 years old with predominantly low-risk pathological features and good general health status (median Karnofsky index = 100%), tumor ≤ 2 cm (69.6%), low to intermediate grade (78.1%), negative axillary nodes (pN0; 73.4%), and positive estrogen receptor (92.2%). Further patient, tumor, and perioperative characteristics are depicted in Tables 1, 2, and 3.

**Table 1:** Patient characteristics, *n* = 128

Age (years)	61.7 ± 12.1
Menopausal status	
*Premenopausal*	18 (14.1%)
*Postmenopausal*	110 (85.9%)
Nationality	
*German*	103 (80.5%)
*Other*	25 (19.5%)
Body mass index (BMI), kg/m^2^	26.4 ± 5.3
Bra size (cup)	
*A*	13 (10.2%)
*B*	38 (29.7%)
*C*	34 (26.6%)
*D*	21 (16.4%)
*E*	4 (3.1%)
*F*	3 (2.3%)
*I*	1 (0.8%)
*Not available*	14 (10.9%)
Breast density ACR	
*1*	10 (7.8%)
*2*	84 (65.6%)
*3*	29 (22.7%)
*4*	5 (3.9%)
Prior surgery of the affected breast?	
*Yes*	10 (7.8%)
*No*	118 (92.2%)
Prior radiation in the target area of the affected breast?	
*Yes*	3 (2.3%)
*No*	125 (97.7%)

*n* (%), *ACR* Breast density according to the American College of Radiology

Results are presented as mean value ± standard deviation (SD)

**Table 2:** Tumor characteristics, *n* = 130

Tumor location	
*Right*	52 (40.6%)
*Left*	74 (57.8%)
*Both sides*	2 (1.6%)
Focality	
*Unifocal*	124 (95.4%)
*Bifocal*	4 (3.1%)
*Multicentric*	2 (1.5%)
Histology	
*No special type (NST)*	101 (77.7%)
*Lobular*	27 (20.8%)
*Mucinous*	2 (1.5%)
Grading	
*G1*	25 (19.2%)
*G2*	76 (58.5%)
*G3*	29 (22.3%)
Estrogen receptor status	
*Positive*	119 (91.5%)
*Negative*	11 (8.5%)
Progesterone receptor status	
*Positive*	108 (83.1%)
*Negative*	22 (16.9%)
Her2neu status	
*Positive*	22 (16.9%)
*Negative*	108 (83.1%)
Tumor location (quadrant)	
*Upper-outer*	62 (47.7%)
*Upper-inner*	26 (20.0%)
*Lower-outer*	23 (17.7%)
*Lower-inner*	7 (5.4%)
*Central*	7 (5.4%)
*Not known*	5 (3.8%)
*Distance from nipple-areola complex (cm)*	4.11 ± 3.0
*Sonographic tumor-to-skin distance (cm)*	0.86 ± 0.44
*Sonographic expansion (largest plane, cm)*	1.3 ± 0.78
Clinical tumor size (cT)	
*cT1a*	1 (0.8%)
*cT1b*	28 (21.5%)
*cT1c*	59 (45.4%)
*cT2*	38 (29.2%)
*cT3*	3 (2.3%)
*cT4*	1 (0.8%)
Pathological tumor size after neoadjuvant chemotherapy	
*ypT0*	16 (12.3%)
*ypT1a*	4 (3.1%)
*ypT1b*	1 (0.8%)
*ypT1c*	6 (4.6%)
*ypT2*	5 (3.8%)
*ypT3*	0 (0%)
*ypT4*	1 (0.8%)
Pathological tumor size without neoadjuvant chemotherapy	
*pT1a*	3 (2.3%)
*pT1b*	22 (16.9%)
*pT1c*	39 (30%)
*pT2*	30 (23.1%)
*pT3*	2 (1.5%)
*pT4*	1 (0.8%)
Axillary nodal status, clinical (cN)	
*cN0*	106 (81.5%)
*cN+*	24 (18.5%)
Axillary nodal status, pathological (pN)	
*pN0*	95 (73.1%)
*≥* *pN1*	31 (23.8%)
*pNx (unknown)*	4 (3.1%)
Tumor palpable (by the physician of the breast unit)	
*Yes*	75 (57.7%)
*No*	55 (42.3%)

*n* (%)

Results are presented as mean value ± standard deviation (SD)

**Table 3:** Perioperative characteristics, *n* = 128

Applicator size (diameter in cm; *n* = 30)	
*4*	11 (36.7%)
*4.5*	13 (43.3%)
*5*	6 (20.0%)
Duration of irradiation (min; *n* = 29; one data missing)	35 ± 8
Dose (Gy) applicator surface	
*20*	30 (100%)
Skin spindle excision	
*Yes*	63 (49.2%)
*No*	65 (50.8%)
Intraoperative performance of a displacement flap	
*Yes*	100 (78.1%)
*No*	28 (21.9%)
Drainage insertion into the wound cavity	
*Yes*	127 (99.2%)
*No*	1 (0.8%)
Drainage insertion into the axilla	
*Yes*	54 (42.2%)
*No*	74 (57.8%)
Duration of in-hospital stay (days)	Median 3 (range: 1–10)

*n* (%)

Results are presented as mean value ± standard deviation (SD) or as median together with range

**Fig. 1:**
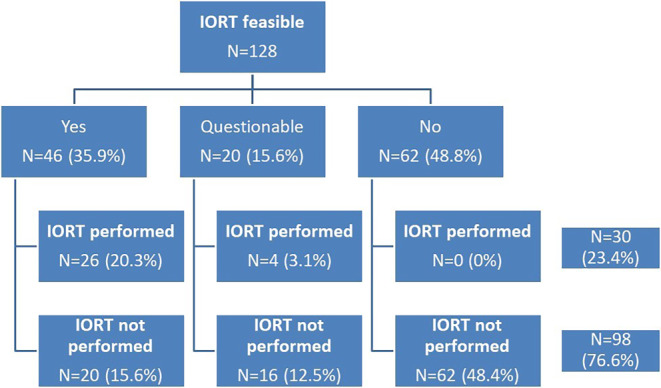
Feasibility and implementation of intraoperative radiotherapy (IORT). (Percentages refer to total sample size [*n* = 128])

Conformity of preoperative *IORT assessment *and *intraoperative outcome *is demonstrated in Fig. 1. A total of 46 (35.9%) women were assigned eligible for IORT from the standpoint of practical feasibility. Of these, 26 finally underwent intraoperative irradiation. Omission of IORT took place due to various reasons in the remaining 20 patients as described below. In 20 of 128 patients (15.6%), IORT was preoperatively considered questionably possible with an IORT being ultimately performed in 4 patients. Finally, due to the reasons listed in Table 4, a total of 62 women (48.4%) were rated unfeasible for IORT. None of the patients who initially appeared unfeasible for IORT ultimately received IORT. In total, 30 (23.4%) out of 128 patients underwent IORT.

**Table 4:** Reasons for preoperatively confirmed unfeasibility of intraoperative radiotherapy (IORT; *n* = 62)

Reason	Frequency	Percent
Minor tumor–skin distance	35	56.5
Oversized tumor cavity	19	30.6
Combination of minor tumor–skin distance and oversized tumor cavity	4	6.5
Multicentric breast cancer	2	3.2
Surgical technique (central segment resection)	1	1.6
Open sampling of a second focal finding	1	1.6

In the case of IORT not performed intraoperatively in the collective of patients “feasible” for IORT or “questionably feasible” according to preoperative assessment, the reasons for omission of IORT were documented by the treating physician after surgery as presented in Table 5.

**Table 5:** Reasons for “IORT not performed” (*n* = 36)

Reason	Frequency	Percent
Minor tumor–skin distance	16	44.4
Oversized tumor cavity	8	22.2
Combination of minor tumor-to-skin distance and oversized tumor cavity	3	8.3
Unclear	2	5.6
No tumor board recommendation for IORT	3	8.3
Second focal finding	1	2.8
Refusal of the patient to undergo IORT	1	2.8
Missing clip in the histopathological resectate	1	2.8
Resection margins not being reliably free in the intraoperative frozen section	1	2.8

*IORT* intraoperative radiotherapy

The preoperative evaluation of patients to be *feasible for IORT* correlated significantly with small tumor size according to both clinical and pathological examinations: clinical tumor size according to TNM classification (cT) by ultrasound (*p* = 0.0003), sonographic tumor size using the largest tumor diameter (cm; *p* = 0.0043) as well as tumor size (largest tumor diameter) according to the histopathological examination (cm; *p* = 0.0056).

A statistically significant association for the preoperative classification “feasible for IORT” was also found for large tumor-to-skin distance (cm; *p* < 0.0001).

As observed for preoperative assessment of feasibility for IORT, also *implementation of IORT* correlated significantly with small tumor size according to clinical assessment via ultrasound (cT; *p* = 0.0035), sonographic tumor size using the largest tumor diameter (cm; *p* = 0.0110), and tumor size (largest tumor diameter) according to histopathological examination (cm; *p* = 0.0095). A large tumor-to-skin distance (cm) was also significantly associated with an IORT being implemented (*p* = 0.0016).

A closer look at our data reveals that of 83 patients with preoperatively sonographically measured tumor–skin distance ≤ 1 cm, IORT was performed in 16 patients (19.2%). Among the 32 patients with tumor–skin distance > 1 cm, IORT was performed in 12 patients (37.5%). In 13 patients, tumor–skin distance was not documented.

A logistic regression analysis analyzing preoperative feasibility assessment of IORT yielded highly significant test results for the variables “clinical tumor size (cT)” (*p* = 0.0014; OR 3.102) and “tumor–skin distance” measured by ultrasound (*p* < 0.0001; OR 0.026). The AUC of 0.864 represents a fairly good model. With each increasing cT level the likelihood risk of IORT to be considered unfeasible triples. In contrast, with each cm increase in tumor-to-skin distance the probability that IORT is considered unfeasible decreases by 97%.

An individual probability calculation of IORT to be feasible can be estimated using the following formula:

$$P\left(\text{IORT feasible}\right)=1-\frac{\exp \left(2.4962+1.1320\times \mathrm{cT}-3.6518\times \text{distance}\right)}{1+\exp \left(2.4962+1.1320\times \mathrm{cT}-3.6518\times \text{distance}\right)}$$

When using this formula, according to preoperative diagnostics “distance” has to be replaced by tumor-to-skin distance in cm, while the clinical tumor stadium “cT” should be replaced by the following numbers: −1 for cT1a, 0 for cT1b, 1 for cT1c, 2 for cT2, 3 for cT3, and 4 for cT4.

As depicted in Fig. 1, there is a high level of concordance between preoperative assessment of patients rated unfeasible or questionably feasible for IORT and nonimplementation of intraoperative irradiation (78 of 82 patients, 95.1%). Applying the above stated formula reveals an IORT probability of 19% for patients rated unfeasible and only 28% for women rated questionably feasible, respectively.

The most common reasons for omission of IORT contrary to the preoperative evaluation “IORT feasible” (*n* = 20) were insufficient tumor-to-skin distance (*n* = 8, 40%) and oversized wound cavity (*n* = 6, 30%). In 3 cases, the tumor board did not recommend IORT, which was therefore not planned and performed (*n* = 3, 15%). Further reasons with one case each were missing clip in the histopathological specimen, intraoperative identification of a second focal finding, and omission of IORT for an unknown reason.

In patients who appeared to be eligible for IORT before surgery, irradiation was performed in only 56.5% (26 of 46 women). This fairly matched the calculated probability of 61% for these patients according to our formula.

Due to this correlation, we had a closer look at the 20 patients in whom IORT was planned and in the end not accomplished. In 14 women we identified anatomico-surgical parameters to be the reason why irradiation was omitted:

In 8 cases an insufficient tumor-to-skin distance was the reason for omission of IORT. In this subgroup preoperative sonographic tumor-to-skin distance was 1.025 cm on average, which has to be considered rather small but (on average) sufficient. However, in one of these patients a cT2 tumor had only 0.5 cm distance to the skin according to preoperative ultrasound examination, which made IORT intraoperatively impracticable. In retrospect, this case should have been classified as unsuitable for IORT preoperatively. This estimate is also strongly supported using the above stated formula with a probability of 5% for suitability of IORT. In 2 cases, each with a cT1c tumor which had a distance of 0.8 and 1 cm to the skin, IORT could not be performed because the surgery resulted in too little skin coverage. Probability for IORT being suitable in these cases was 33 and 51%, respectively. Furthermore, in 4 cases resection was performed as a consequence of frozen section or according to intraoperative sonographic and palpatory findings, which resulted in an insufficient tumor–skin distance, so that IORT could not be performed. In this subgroup, the average probability for IORT being suitable was only 49% (*P* = 0.255–0.5059). Finally, there was one case in which, despite a tumor–skin distance of 2 cm according to preoperative ultrasound in a cT1b tumor, IORT was not performed. Using our formula, the probability for feasibility of IORT was 99%. In this patient a small breast size with insufficient soft tissue coverage around the excision cavity led to the decision not to perform IORT.

Concerning omission of IORT despite a positive preoperative assessment due to an excessively large tumor cavity intraoperatively, we identified 6 patients: In 1 patient, IORT was omitted for a cT1b tumor with a wound cavity that was too large for IORT to be performed. Follow-up resections were accomplished in 5 patients for the following reasons: In 3 cases frozen section revealed tumor residues along the resection margins, in one woman due to a macroscopically conspicuous area in the tumor environment and in one patient because of a missing clip in the main sample radiography. In all of these cases follow-up resection led to enlargement of the tumor cavity and thus to omission of IORT. The probability *P *for IORT to be suitable in these patients averaged 81% (*P* = 0.506–0.976) according to our formula. These findings show that besides consideration of clinical measures IORT is susceptible not to be performed due to unfavorable histological findings with the necessity of follow-up resection.

## Discussion

Looking at the currently existing guidelines of the updated ASTRO consensus status, the use of IORT outside clinical trials should be restricted to women who otherwise meet “suitable” criteria for partial breast irradiation. According to the ASTRO, application of IORT as APBI can be considered for women at the age of ≥ 50 years with unicentric, pT1, pN0, nonlobular invasive breast cancer, positive estrogen receptor status, negative margins by at least 2 mm as well as DCIS when meeting specific criteria [14]. The GEC-ESTRO recommends performing APBI only for a low-risk group that includes women aged at least 50 years with unicentric, unifocal, pT1–2 (≤ 3 cm), pN0, nonlobular invasive breast cancer with any hormone receptor status and negative margins by at least 2 mm [13]. Recently the American Society of Breast Surgeons (ASBS) has published another consensus guideline on APBI for the treatment of breast cancer. The criteria include women ≥ 45 years with unifocal or multifocal tumor (if total span of tumor is ≤ 3 cm), pTis, pT1–2 (≤ 3 cm), pN0, all invasive subtypes and DCIS, any hormone receptor status, no invasive tumor on ink, negative margins by at least 2 mm for DCIS and women without BRCA genetic mutation or other genetic mutation that confers an increased risk of breast cancer [22]. According to the German Society of Radiation Oncology (DEGRO), outside of clinical trials, partial breast irradiation should only be performed under the following conditions: age ≥ 50 years with any invasive carcinoma (any grade) or DCIS, G1–2, tumor size: ≤ 3 cm (Tis, T1–T2), unifocal and unicentric DCIS or breast cancer, negative resection margins for invasive cancer by at least 2 mm, for invasive lobular histology or DCIS at least 5 mm, L0, V0, pN0/pNmi. The following should be considered as contraindications for APBI: stage IIB–IV breast cancer, resection margins that cannot be microscopically assessed, extensive intraductal component (EIC), Paget’s disease or pathological skin involvement, age ≤ 40 years, triple-negative or HER2-positive phenotype and neoadjuvant chemotherapy in treatment history [23]. With regard to IORT, the DEGRO expert panel recommends that the 50 kV system (INTRABEAM®) should preferentially be used in the context of clinical trials. When used, it should be restricted to women with all of the following criteria: invasive cancer, aged > 70 years, tumor < 2 cm, resection margins > 2 mm, grade 1–2, pN0, ER positive, HER2 negative, L0, V0, and EIC negative [23].

Despite minor differences, all of the aforementioned guidelines reflect the results of underlying randomized controlled trials, selecting patients with a low probability of local recurrence.

Furthermore, a tumor bed boost by single intraoperative irradiation with IORT in addition to whole breast irradiation is recommended for premenopausal women or for postmenopausal women at higher risk of local recurrence (> T1, G3, Her2-positive, triple negative, EIC) [21].

Since these recommendations do not take into account specific preoperative diagnostic or surgical parameters apart from tumor size, it is not surprising that the omission rate of intended IORT seems to be rather high:

Sperk et al. were able to show that after verifying the suitability for IORT as APBI according to the ESTRO and ASTRO consensus statement in 1108 cases (leading to a suitability of 34.2% according to ESTRO and 15.8% according to the ASTRO consensus statement) not even half (ESTRO 41.4%, ASTRO 45.1%) were ultimately treated with IORT as APBI [24].

The reasons why a planned IORT could ultimately not be performed were investigated in detail by Tuschy et al. [25]: they could show that in 18.5% of 297 analyzed patients a preoperatively planned IORT could not be performed intraoperatively for the following reasons: small tumor–skin distance (< 5–10 mm) in 35.1%, oversized dissection cavities in 24.6% or a combination of both in 14% [25]. These retrospective findings go well in line with the results of the present investigation according to which an insufficient tumor–skin distance (40%) and an oversized wound cavity (30%) are the main reasons for omission of intended IORT.

The results of a study by Kim et al., in which surgical factors associated with cancellation of planned IORT were evaluated, match our results fairly, as authors found the main reasons for omission of IORT to be an oversized tumor cavity in 33.3% due to repeated positive margins in frozen section that required follow-up resections. Conversion to total mastectomy in 16.7%, an insufficient cavity-to-skin distance in 15.6%, and cavity geometry unfit for IORT in 6.7% were other reasons for omission of IORT. Authors found that the presence of a satellite tumor was a strong predictor for IORT cancellation (*p* < 0.0001) [30].

These results are conclusive with the findings of Jung et al. according to whom multifocal disease as well as concomitant carcinoma in situ were identified as predisposing factors for positive margins, consecutively leading to higher resection rates among patients undergoing BCS [31]. Follow-up resections in turn lead to an enlargement of the tumor cavity.

Considering the current data regarding omission of IORT an oversized tumor cavity as well as a minor tumor-to-skin distance seem to be the most frequent reasons why a planned IORT is not accomplished. Correlating with these measures, small tumor size and larger tumor-to-skin distance showed a clear prediction for accomplishment of planned IORT in our prospectively designed study, so that we were able to generate a formula with which the individual probability of IORT feasibility can be calculated.

Our aforementioned cases, presented in the results section, underline the pivotal relevance of tumor–skin distance in IORT planning as well as the necessity of furthermore considering clinical parameters such as breast size and breast–tumor ratio.

From our point of view, a sonographic distance of at least 1 cm is necessary for planning an IORT. This is supported by our data that IORT was performed in less than 20% of cases with a preoperative sonographic distance of < 1 cm. If the sonographic distance is less than 1 cm, a definitive distance of less than 1 cm can be assumed after tumor resection. Here, the risk of radiation-induced toxicity increases significantly.

Since our formula does not consider intraoperative findings (e.g., follow-up resection due to frozen section) nor clinical parameters beyond tumor size and tumor–skin distance that can potentially affect the applicability of IORT, predictive quality is limited. Another limitation of our study is the lack of predefined selection criteria regarding the assessment for feasibility of IORT (especially tumor–skin distance and tumor size). In patients who were clinically assessed as feasible for IORT, IORT was performed in only 56.5%. The insufficient preselection criteria also led to a poorer performance of our model.

Despite these limitations, we consider our formula to be an easy tool in order to more precisely identify patients eligible for IORT under real-life conditions, taking preoperatively assessed sonographic measures into account. This enables the treating physician and patient to select and plan IORT more precisely and to reduce the burden associated with planned but not accomplished treatments. Besides the organizational benefits, patient satisfaction is presumably improved with accurate implementation of preoperative counseling.

While tumor-to-skin distance besides tumor size seems to be of major relevance in this context, also further clinical parameter such as tumor–breast ratio, tumor location should always be considered when planning IORT. Furthermore, it is apparent that unsuitable histological findings intraoperatively can lead to omission of IORT. This underlines the importance of multimodal interdisciplinary imaging (e.g., ultrasound, mammography, magnetic resonance imaging [MRI]) before surgery, to best possibly identify parameters in advance that might compromise the performance of IORT.

Planning IORT taking these measures besides guideline recommendations into account should improve IORT implementation rates.

## Conclusion

Our results show that in addition to recommendations of existing guidelines including tumor size, especially preoperative measurement of tumor–skin distance via ultrasound should be considered in the planning of intraoperative radiotherapy (IORT) in early breast cancer surgery.

## Appendix

**Table 6:** Preoperative questionnaire

Patient code:	Date:
*Do you consider the above-mentioned patient feasible for IORT?*	
□ yes	
□ questionable (planning for IORT, but feasibility uncertain)	
□ no	
*If no, why?*	
□ insufficient tumor–skin distance	
□ large tumor cavity	
□ a combination of both	
□ others, please specify	
Reporting physician (name, signature):	

## References

[1] (2021) Zentrum für Krebsregisterdaten am Robert-Koch-Institut. https://www.krebsdaten.de/Krebs/DE/Content/Krebsarten/Brustkrebs/brustkrebs_node.html. Accessed 19 Apr 2021

[2] Rojas K, Stuckey A (2016). Breast cancer epidemiology and risk factors. Clin Obstet Gynecol.

[3] S3 (2020) Interdisziplinäre S3-Leitlinie für die Früherkennung, Diagnostik, Therapie und Nachsorge des Mammakarzinoms. Langversion 4.3 – Februar 2020 AWMF-Registernummer: 032-045OL https://www.awmf.org/uploads/tx_szleitlinien/032-045OLl_S3_Mammakarzinom_2020-02.pdf.

[4] Sedlmayer F, Sautter-Bihl ML, Budach W, Dunst J, Fastner G, Feyer P (2013). DEGRO practical guidelines: radiotherapy of breast cancer I: radiotherapy following breast conserving therapy for invasive breast cancer. Strahlenther Onkol.

[5] Shah C, Badiyan S, Wilkinson BJ, Vicini F, Beitsch P, Keisch M (2013). Treatment efficacy with accelerated partial breast irradiation (APBI): final analysis of the American Society of Breast Surgeons MammoSite((R)) breast brachytherapy registry trial. Ann Surg Oncol.

[6] Polgar C, Fodor J, Major T, Sulyok Z, Kasler M (2013). Breast-conserving therapy with partial or whole breast irradiation: ten-year results of the Budapest randomized trial. Radiother Oncol.

[7] Pashtan IM, Recht A, Ancukiewicz M, Brachtel E, Abi-Raad RF, D’Alessandro HA (2012). External beam accelerated partial-breast irradiation using 32 gy in 8 twice-daily fractions: 5-year results of a prospective study. Int J Radiat Oncol Biol Phys.

[8] Lemanski C, Azria D, Gourgou-Bourgade S, Ailleres N, Pastant A, Rouanet P (2013). Electrons for intraoperative radiotherapy in selected breast-cancer patients: late results of the Montpellier phase II trial. Radiat Oncol.

[9] Veronesi U, Orecchia R, Maisonneuve P, Viale G, Rotmensz N, Sangalli C (2013). Intraoperative radiotherapy versus external radiotherapy for early breast cancer (ELIOT): a randomised controlled equivalence trial. Lancet Oncol.

[10] Vaidya JS, Joseph DJ, Tobias JS, Bulsara M, Wenz F, Saunders C (2010). Targeted intraoperative radiotherapy versus whole breast radiotherapy for breast cancer (TARGIT-A trial): an international, prospective, randomised, non-inferiority phase 3 trial. Lancet.

[11] Vaidya JS, Wenz F, Bulsara M, Tobias JS, Joseph DJ, Keshtgar M (2014). Risk-adapted targeted intraoperative radiotherapy versus whole-breast radiotherapy for breast cancer: 5-year results for local control and overall survival from the TARGIT-A randomised trial. Lancet.

[12] Orecchia R, Veronesi U, Maisonneuve P, Galimberti VE, Lazzari R, Veronesi P (2021). Intraoperative irradiation for early breast cancer (ELIOT): long-term recurrence and survival outcomes from a single-centre, randomised, phase 3 equivalence trial. Lancet Oncol.

[13] Polgar C, Van Limbergen E, Potter R, Kovacs G, Polo A, Lyczek J (2010). Patient selection for accelerated partial-breast irradiation (APBI) after breast-conserving surgery: recommendations of the Groupe Europeen de Curietherapie-European Society for Therapeutic Radiology and Oncology (GEC-ESTRO) breast cancer working group based on clinical evidence (2009). Radiother Oncol.

[14] Correa C, Harris EE, Leonardi MC, Smith BD, Taghian AG, Thompson AM (2017). Accelerated partial breast irradiation: Executive summary for the update of an ASTRO evidence-based consensus statement. Pract Radiat Oncol.

[15] Vaidya JS, Bulsara M, Baum M, Wenz F, Massarut S, Pigorsch S (2021). New clinical and biological insights from the international TARGIT-A randomised trial of targeted intraoperative radiotherapy during lumpectomy for breast cancer. Br J Cancer.

[16] Vaidya JS, Bulsara M, Baum M, Tobias JS, authors T-At (2021). Single-dose intraoperative radiotherapy during lumpectomy for breast cancer: an innovative patient-centred treatment. Br J Cancer.

[17] Vaidya JS, Bulsara M, Baum M, Wenz F, Massarut S, Pigorsch S (2020). Long term survival and local control outcomes from single dose targeted intraoperative radiotherapy during lumpectomy (TARGIT-IORT) for early breast cancer: TARGIT-A randomised clinical trial. BMJ.

[18] Vavassori A, Tagliaferri L, Vicenzi L, D’Aviero A, Ciabattoni A, Gribaudo S (2020). Practical indications for management of patients candidate to Interventional and Intraoperative Radiotherapy (Brachytherapy, IORT) during COVID-19 pandemic—A document endorsed by AIRO (Italian Association of Radiotherapy and Clinical Oncology) Interventional Radiotherapy Working Group. Radiother Oncol.

[19] Blank E, Kraus-Tiefenbacher U, Welzel G, Keller A, Bohrer M, Sutterlin M (2010). Single-center long-term follow-up after intraoperative radiotherapy as a boost during breast-conserving surgery using low-kilovoltage x-rays. Ann Surg Oncol.

[20] Pez M, Keller A, Welzel G, Abo-Madyan Y, Ehmann M, Tuschy B (2020). Long-term outcome after intraoperative radiotherapy as a boost in breast cancer. Strahlenther Onkol.

[21] Arbeitsgemeinschaft Gynäkologische Onkologie EV Guidelines Breast Version 2020.1D. Adjuvante Strahlentherapie. https://www.ago-online.de/fileadmin/ago-online/downloads/_leitlinien/kommission_mamma/2022/Einzeldateien/AGO_2022D_13_Adjuvante_Strahlentherapie.pdf. Accessed 13 Jan 2023

[22] The American Society of Breast Surgeons (2018) Consensus guideine on accelerated partial breast irradiation. https://www.breastsurgeons.org/docs/statements/Consensus-Statement-for-Accelerated-Partial-Breast-Irradiation.pdf. Accessed 16 Apr 2021

[23] Strnad V, Krug D, Sedlmayer F, Piroth MD, Budach W, Baumann R (2020). DEGRO practical guideline for partial-breast irradiation. Strahlenther Onkol.

[24] Sperk E, Astor D, Keller A, Welzel G, Gerhardt A, Tuschy B (2014). A cohort analysis to identify eligible patients for intraoperative radiotherapy (IORT) of early breast cancer. Radiat Oncol.

[25] Tuschy B, Berlit S, Nasterlack C, Tome K, Blank E, Wenz F (2013). Intraoperative radiotherapy of early breast cancer using low-kilovoltage x-rays-reasons for omission of planned intraoperative irradiation. Breast J.

[26] Vaidya JS, Baum M, Tobias JS, D’Souza DP, Naidu SV, Morgan S (2001). Targeted intra-operative radiotherapy (Targit): an innovative method of treatment for early breast cancer. Ann Oncol.

[27] Vaidya JS, Baum M, Tobias JS, Morgan S, D’Souza D (2002). The novel technique of delivering targeted intraoperative radiotherapy (Targit) for early breast cancer. Eur J Surg Oncol.

[28] Kraus-Tiefenbacher U, Steil V, Bauer L, Melchert F, Wenz F (2003). A novel mobile device for intraoperative radiotherapy (IORT). Onkologie.

[29] Good Clinical Practice Network Clinical Trial NCT02290782. TARGIT-C(Consolidation) Prospective Phase IV Study of IORT in Patients With Small Breast Cancer (TARGIT-C). https://ichgcp.net/clinical-trials-registry/NCT02290782. Accessed 13 Jan 2023

[30] Kim JW, Cho Y, Choi J, Ahn SG, Jeong J, Lee IJ (2019). Redefining eligibility by analyzing canceled Intraoperative radiotherapy as a boost for patients undergoing breast-conserving treatment. Ann Surg Oncol.

[31] Jung W, Kang E, Kim SM, Kim D, Hwang Y, Sun Y (2012). Factors associated with re-excision after breast-conserving surgery for early-stage breast cancer. J Breast Cancer.

